# Systematic Review of Anastomotic Leakage Rate According to an International Grading System Following Anterior Resection for Rectal Cancer

**DOI:** 10.1371/journal.pone.0075519

**Published:** 2013-09-25

**Authors:** Zhi-Jie Cong, Liang-Hao Hu, Zheng-Qian Bian, Guang-Yao Ye, Min-Hao Yu, Yun-He Gao, Zhao-Shen Li, En-Da Yu, Ming Zhong

**Affiliations:** 1 Department of Colorectal Surgery, Ren Ji Hospital, School of Medicine, Shanghai Jiao Tong University, Shanghai, China; 2 Digestive Endoscopy Center, Changhai Hospital, Second Military Medical University, Shanghai, China; University of Aberdeen, United Kingdom

## Abstract

**Background:**

A generally acceptable definition and a severity grading system for anastomotic leakages (ALs) following rectal resection were not available until 2010, when the International Study Group of Rectal Cancer (ISGRC) proposed a definition and a grading system for AL.

**Methods:**

A search for published data was performed using the MEDLINE database (2000 to December 5, 2012) to perform a systematic review of the studies that described AL, grade AL according to the grading system, pool data, and determine the average rate of AL for each grade after anterior resection (AR) for rectal cancer.

**Results:**

A total of 930 abstracts were retrieved; 40 articles on AR, 25 articles on low AR (LAR), and 5 articles on ultralow AR (ULAR) were included in the review and analysis. The pooled overall AL rate of AR was 8.58% (2,085/24,288); the rate of the asymptomatic leakage (Grade A) was 2.57%, that of AL that required active intervention without relaparotomy (Grade B) was 2.37%, and that of AL that required relaparotomy (Grade C) was 5.40%. The pooled rate of AL that required relaparotomy was higher in AR (5.40%) than in LAR (4.70%) and in ULAR (1.81%), which could be attributed to the higher rate of protective defunctioning stoma in LAR (40.72%) and ULAR (63.44%) compared with that in AR (30.11%).

**Conclusions:**

The new grading system is simple that the ALs of each grade can be easily extracted from past publications, therefore likely to be accepted and applied in future studies.

## Introduction

Anastomotic leakage (AL) is the commonest major complication after rectal cancer surgery and can result in the need for additional surgery, prolonged hospital stays, increased morbidity and mortality and possibly a poorer oncological prognosis [1]. AL occurred in 1% to 21% of individuals with anterior resection (AR) for rectal cancer, as reported in several clinical trials [2-4]. However, the reported rate of AL varies worldwide. This variation can in part be attributed to the lack of a generally acceptable definition and grading of the severity of AL until 2010. This definition and grading system for AL was proposed by the International Study Group of Rectal Cancer (ISGRC) [5]. This system has since been used by some groups to describe AL in the current literature [6,7]. Our study aims to review systematically the studies that describe AL, grade AL according to the grading system proposed by ISGRC. We will then pool the data and determine the average rate of AL for each grade after AR for rectal cancer surgery. Given the impact that total mesorectal excision and stapling devices have had on AL, only studies using these techniques after the year 2000 will be included in this review. In the absence of evidence based on prospective trials of grading AL, this pooled systematic analysis could underpin the current evidence base and thus provide relatively definitive information on this common and potentially life threatening complication.

## Methods

### Definition and severity grading of AL

ISGRC defined AL as a defect in the intestinal wall integrity at the colorectal or coloanal anastomotic site (including suture and staple lines of neorectal reservoirs), which leads to communication between the intra- and extraluminal compartments. A pelvic abscess close to the anastomosis is also considered as AL. The group suggested grading the severity of AL based on its effect on the patients’ clinical management. Grade A was defined as a leakage that requires no active therapeutic intervention. Grade B was defined as a leakage that requires active therapeutic intervention but can be managed without relaparotomy. Grade C was defined as a leakage that requires relaparotomy [5].

### Literature search and selection strategy

Relevant studies published between January 2000 and December 2012 were identified from the search of the Medline databases. The following search terms were used: (rectum OR rectal OR proctectomy) AND (leakage OR failure OR integrity OR insufficiency OR breakdown OR defect OR separation OR dehiscence). Additional relevant articles were then obtained using the citations in the publications identified by the initial search.

Publications in English language that met the following inclusion criteria—either (i, ii, and iii) or (i, ii, and iv)—were selected: (i) availability of laparotomic or laparoscopic sphincter-saving resection for rectal cancer; (ii) availability of data and an incidence rate of AL; (iii) subsequent management of AL, including conservative treatment or relaparotomy reported; and (iv) data on patients with routine imaging studies such as contrast enema after the initial operation or before the DS was closed.

Studies that considered preoperative chemoradiation therapy as the study object were excluded from the analysis, as well as those that used experimental surgical techniques such as single-access laparoscopic or robot-assisted surgery. Two authors (C.ZJ, and H.LH) independently reviewed each of the included studies and extracted data from them. Any discrepancies were resolved by discussion. To increase the sensitivity of the search strategy, the reference lists of the retrieved literature were cross searched manually for additional relevant publications.

**Figure 1:**
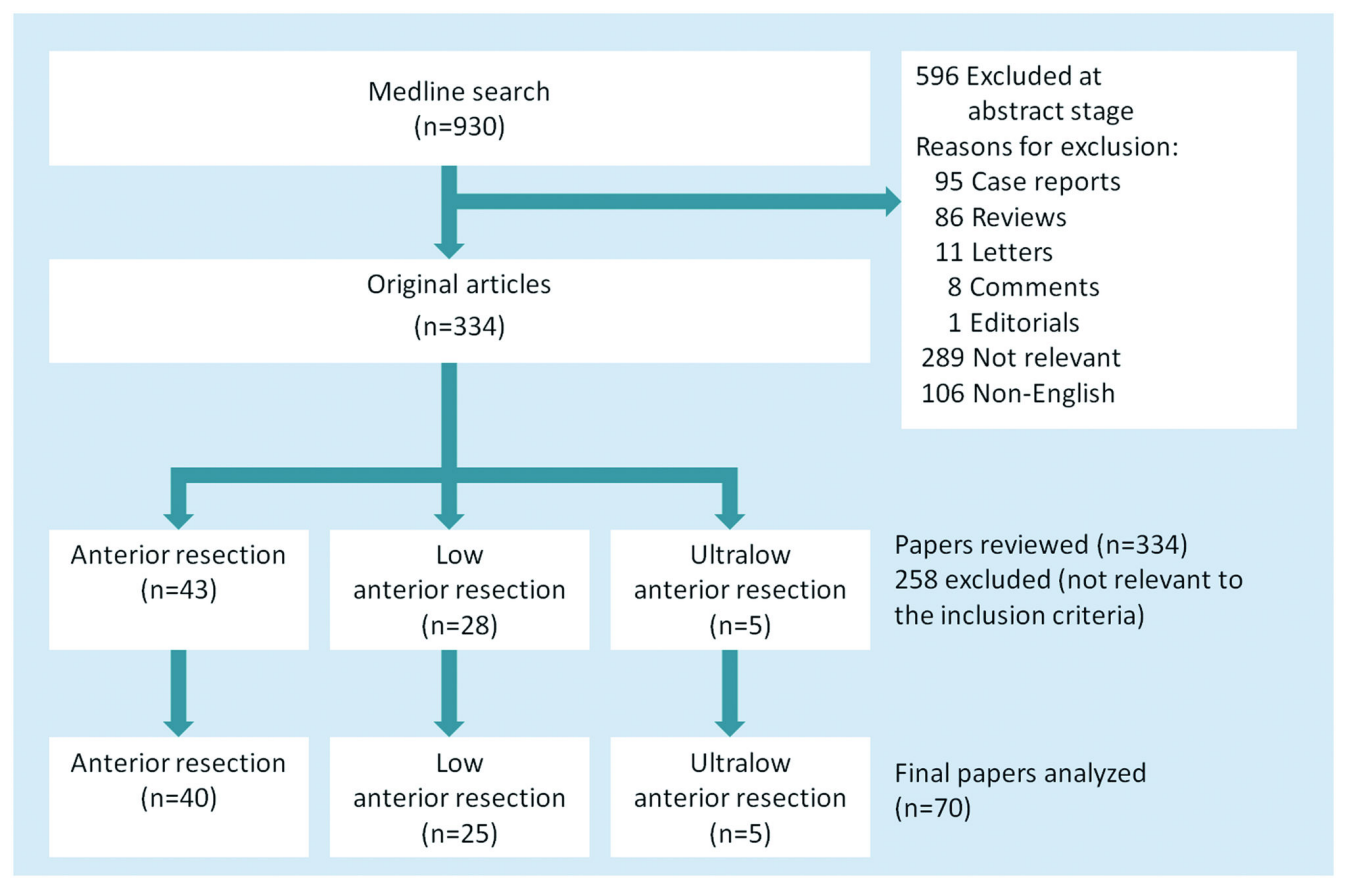
Literature search, review, and analysis.

### Data extraction and analysis strategy

We used the number of ALs based on their definition in the studies. Patients with AL were divided into three grades according to the definition of the grading proposed by ISGRC. The selected studies were divided into three groups: AR, low AR (LAR), and ultralow AR (ULAR). Because the studies on AR are the most common and representative in the current literature, which also include patients with LAR and ULAR, we chose the AR studies as the main part for the analysis and discussion in our research. The pooled rates for each grade of AL extracted from the studies on AR were presented. Then, the data from AR were compared with the data from LAR and ULAR. The data of the AR studies from different countries were also compared. When available, the rate of defunctioning ileostomy/colostomy was also extracted from the studies for comparison.

### Statistical analysis

Statistical analysis of the relative frequencies was conducted with the chi-squared test using the Statistical Package for Windows, version 13.0 (SPSS, Chicago, Illinois, USA). A two-sided *P* value of <0.05 was considered as statistically significant.

## Results

### Bibliometrics

A total of 930 abstracts were retrieved from Medline from 2000 to December 5, 2012. Among these, 106 non-English articles and 289 non-relevant English articles with no or minimal association with AL were excluded. Some additional 201 articles were excluded after further examination of the downloaded abstracts based on the criteria shown in Figure 1. Thus, 334 full papers were examined. Among these, 258 were rejected as irrelevant, which left 43 studies on AR, 28 studies on LAR, and 5 studies on ULAR. Further review of the full papers revealed that three studies on AR patients [8-10] and one study on LAR patients [11] were conducted in the same hospital and duplicated the time of the patients in the other four studies; two other studies did not provide a conclusion whether their patients all underwent LAR [12,13]. Thus, the final analysis included 70 studies: 40 on AR patients [2-4,6,14-49], 25 on LAR patients [7, 50-73], and 5 on ULAR patients with AL [74-78] (Figure 1).

**Figure 2:**
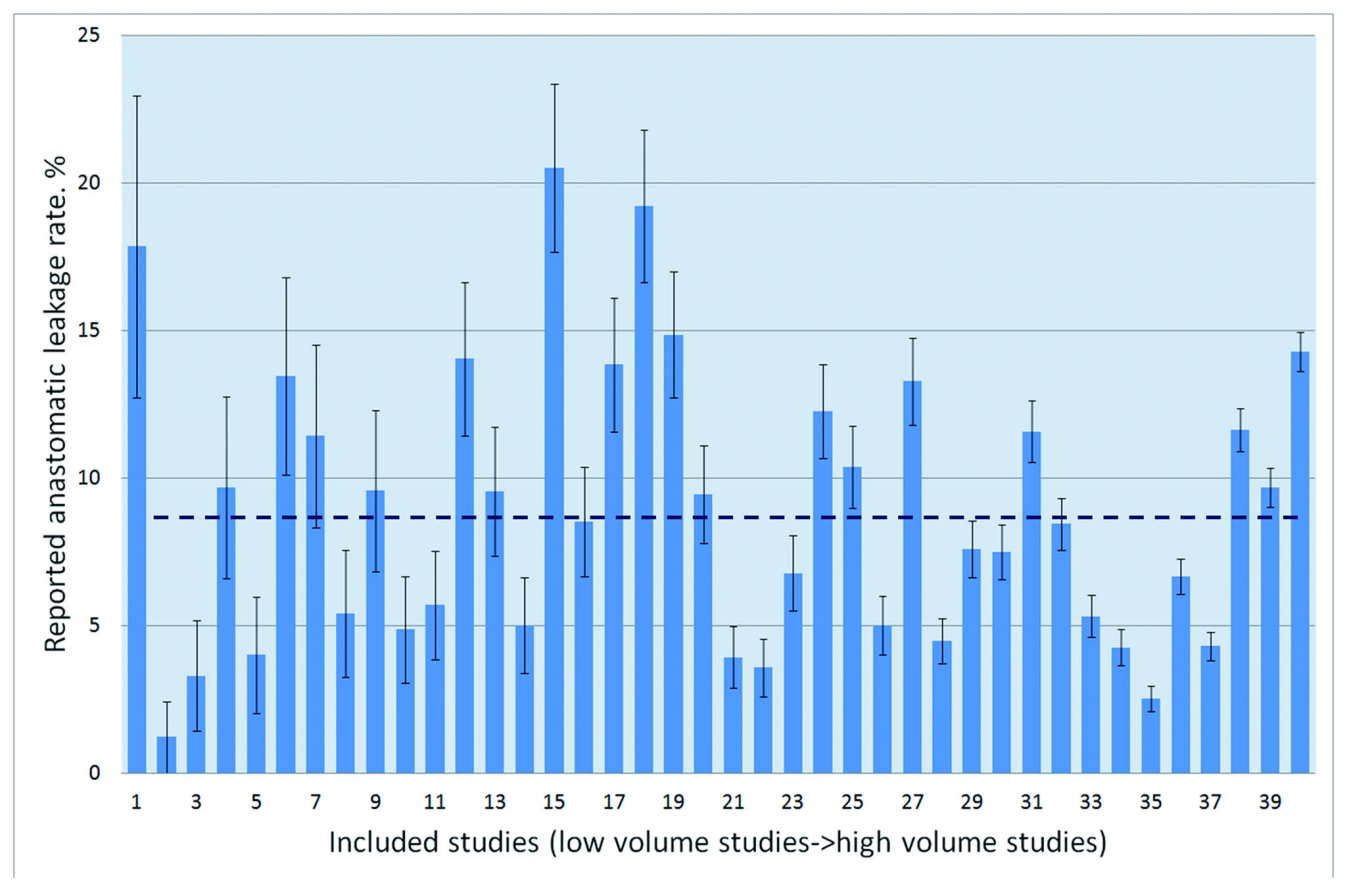
AL rates reported by the included studies on AR ranked by volume.

**Figure 3:**
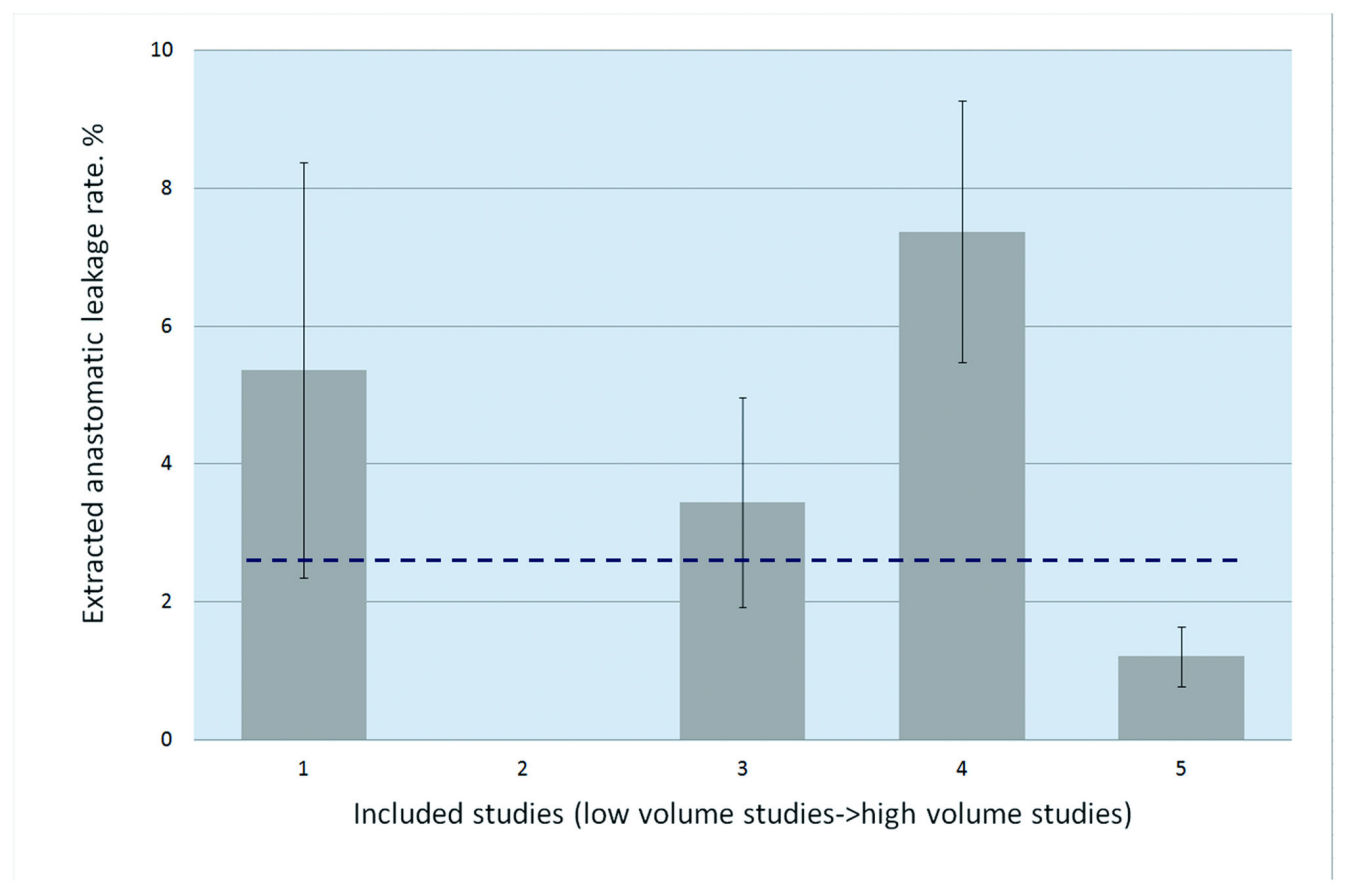
Extracted rates of Grade A leakage ranked by volume.

### Pooled rate of AL for each grade in patients with AR

#### Incidence of reported AL

A total of 40 studies that addressed the rate of AL after AR were analyzed. Four randomized controlled trials were performed, and the numbers of non-randomized prospective and retrospective clinical trials were 13 and 23, respectively. The included studies had a total population of 24,288 patients. The sample sizes of the studies varied from 56 to 2,729 patients.

AL was described in 32 of the 40 (80%) studies. Twenty-seven studies provided a detailed description of the definition of AL, most of which consisted of a clinical suspicion based on the patient’s clinical symptoms, which were subsequently confirmed by endoscopy or imaging studies. Only one author described AL according to the definition proposed by ISGRC [6]. The remaining five studies provided only a limited description.

In the 40 studies, the number of patients confirmed to have anastomotic leaks ranged from 1 to 390, with a total of 2,085. The pooled overall rate of AL was 8.58%. A large variation in the AL rates was observed in the studies; the highest reported AL rate was 20.50%, whereas the lowest was 1.22% (Figure 2).

**Figure 4:**
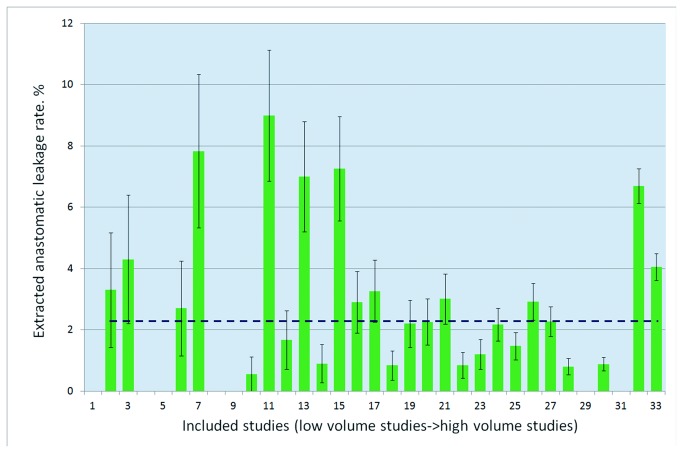
Extracted rates of Grade B leakage ranked by volume.

#### Incidence of Grade A leakage

Grade A AL was defined as an “asymptomatic/radiologic leakage,” which was usually not considered as the object of the study by almost all authors because it required no change in the patient management. Only five studies [3,31,39,45,49] reported that imaging studies such as contrast enema were routinely performed after the initial operation or before the stoma revision to detect a radiological AL, where we obtained the number of events with Grade A leakage. Since the guideline of routine imaging after rectal resection was not available, the timing of imaging among studies was different from each other. So we didn’t give a pooled data of the timing of imaging in this systematic review. The studies showed that 30 out of 1,167 patients with routine imaging studies had a Grade A leakage, and the pooled rate was 2.57%, which varied from 0% to 7.37% (Figure 3).

**Table 1:** Pooled rate of AL for each grade after AR in different countries.

Country(n*)	AL										
	Grade A			Grade B			Grade C			Total	
	Count	%		Count	%		Count	%		Count	%
**Asia**											
China(4)	N/A	N/A		36 (4)	1.76		90 (4)	4.40		126 (4)	6.16
Hong Kong(1)	N/A	N/A		3 (1)	3.30		0 (1)	0		3 (1)	3.30
Japan(5)	N/A	N/A		14 (4)	1.90		51 (5)	2.95		113 (5)	6.53
Korea(7)	N/A	N/A		19 (7)	0.42		197 (7)	4.34		216 (7)	4.76
Taiwan(1)	N/A	N/A		N/A	N/A		37 (1)	3.70		53 (1)	5.31
Total(18)	N/A	N/A		72 (16)	0.97		375 (18)	3.99%		511 (18)	5.43
**Europe**											
Austria(1)	N/A	N/A		4 (1)	0.85		45 (1)	9.53		49 (1)	10.38
Belgium(1)	N/A	N/A		16 (1)	0.88		105 (1)	5.79		121 (1)	6.67
France(2)	19 (2)	5.67		30 (2)	7.94		17 (2)	4.50		66 (2)	17.46
Germany(6)	N/A	N/A		34 (3)	2.39		343 (6)	6.99		606 (6)	12.35
Greece(1)	N/A	N/A		4 (1)	4.30		5 (1)	5.38		9 (1)	9.68
Italy(2)	0 (1)	0		9 (1)	7.83		12 (2)	5.48		25 (2)	11.42
Ireland(1)	N/A	N/A		0 (1)	0		4 (1)	4.00		4 (1)	4.00
Netherlands(2)	N/A	N/A		21 (2)	1.97		93 (2)	8.71		114 (2)	10.67
Norway(2)	3 (1)	5.36		131 (1)	6.69		97 (1)	4.95		238 (2)	11.82
Sweden(3)	N/A	N/A		112 (3)	4.17		182 (3)	6.77		294 (3)	10.93
Total(21)	22 (4)	4.35		361 (16)	3.57		903 (20)	6.59%		1,526 (21)	11.09
**USA**(1)	8 (1)	1.21		9 (1)	0.80		31 (1)	2.75		48 (1)	4.26

* number of reports

**Figure 5:**
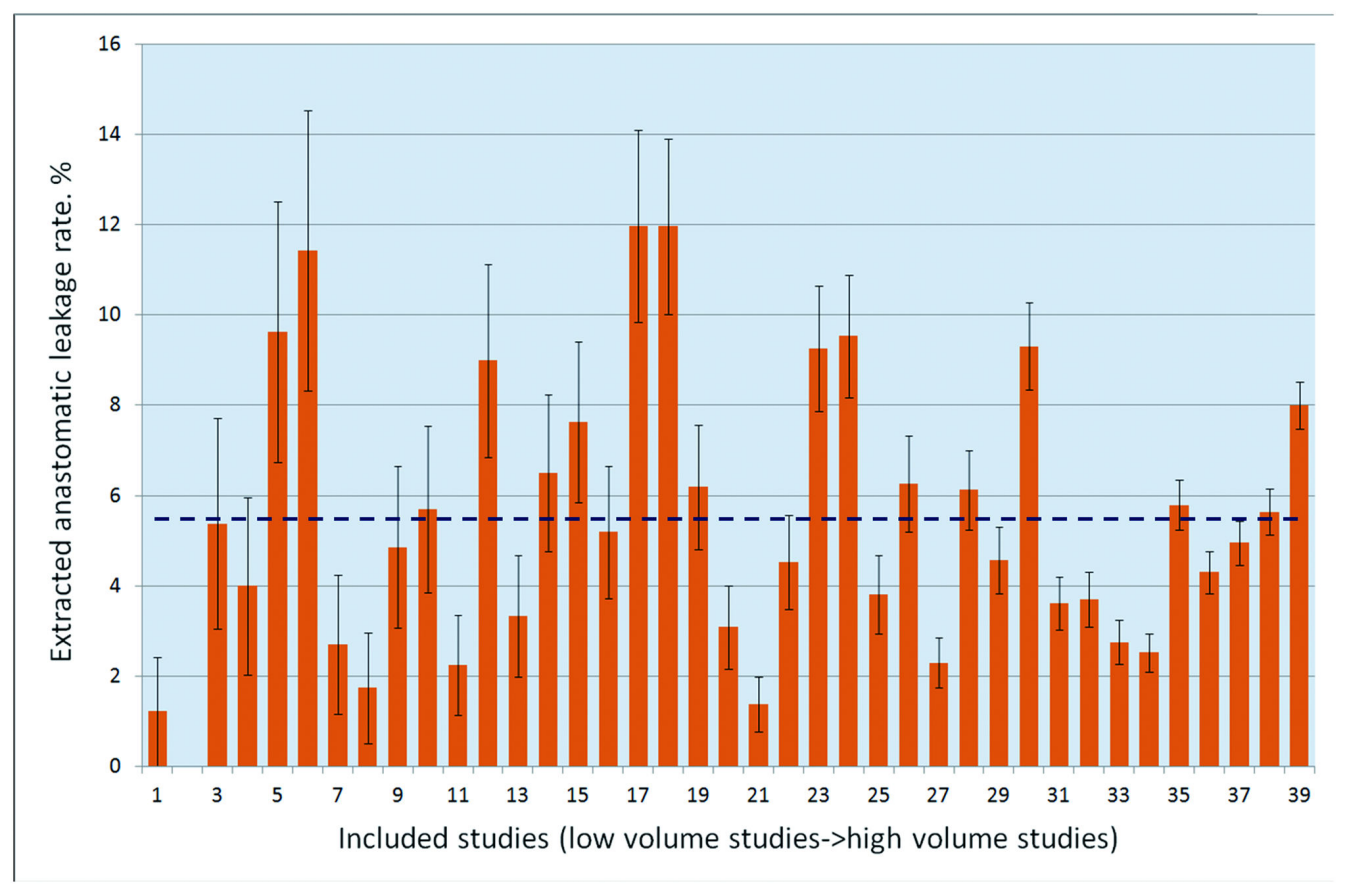
Extracted rates of Grade C leakage ranked by volume.

#### Incidence of Grade B leakage

Based on non-surgical interventions (such as antibiotics) or interventional drainage or transanal lavage described in 33 studies, we extracted the rates of Grade B leakage in these studies. A total of 442 out of 18,647 patients had subsequent Grade B leakage after AR for rectal cancer. The pooled incidence rate of the Grade B leakage was 2.37%, which varied from 0% to 8.99% (Figure 4).

#### Incidence of Grade C leakage

The number of Grade C leakage was extracted from 39 studies. A total of 1,309 Grade C leakage cases were confirmed in this review, which means that 63.08% (1,309/2,075) of the reported ALs after AR for rectal cancer required operative re-intervention. The pooled incidence rate of Grade C leakage was 5.40% (1,309/24,232); the highest was 11.97%, and the lowest was 0% (Figure 5). Twenty-three authors reported surgical procedure of re-intervention for Grade C leakage in their studies. A total of 536 Grade C leakage cases were found in these specific studies, and 480 (89.55%) needed temporary or permanent diverting-stoma construction.

### AL in patients with LAR and ULAR compared with AR

The ALs reported in the studies on LAR and ULAR were also reviewed. Twenty-five studies on LAR matched the inclusion criteria, which had a total population of 4,664 patients. The LAR definition was described in 11 of 25 (44%) studies. Most of the description of the definition consisted of the level of anastomosis or tumor margin, which should be below the peritoneal reflection or from lower than 6 cm to 8 cm from the anal verge. A total of 414 leaks were confirmed; the pooled overall rate of AL was 8.88% (which ranged from 1.89% to 20.59%). The pooled rates of Grades A, B, and C leakages were 1.14% (8/701 from 3 studies), 3.75% (151/4,022 from 22 studies), and 4.70% (219/4,664 from all 25 studies), respectively.

**Figure 6:**
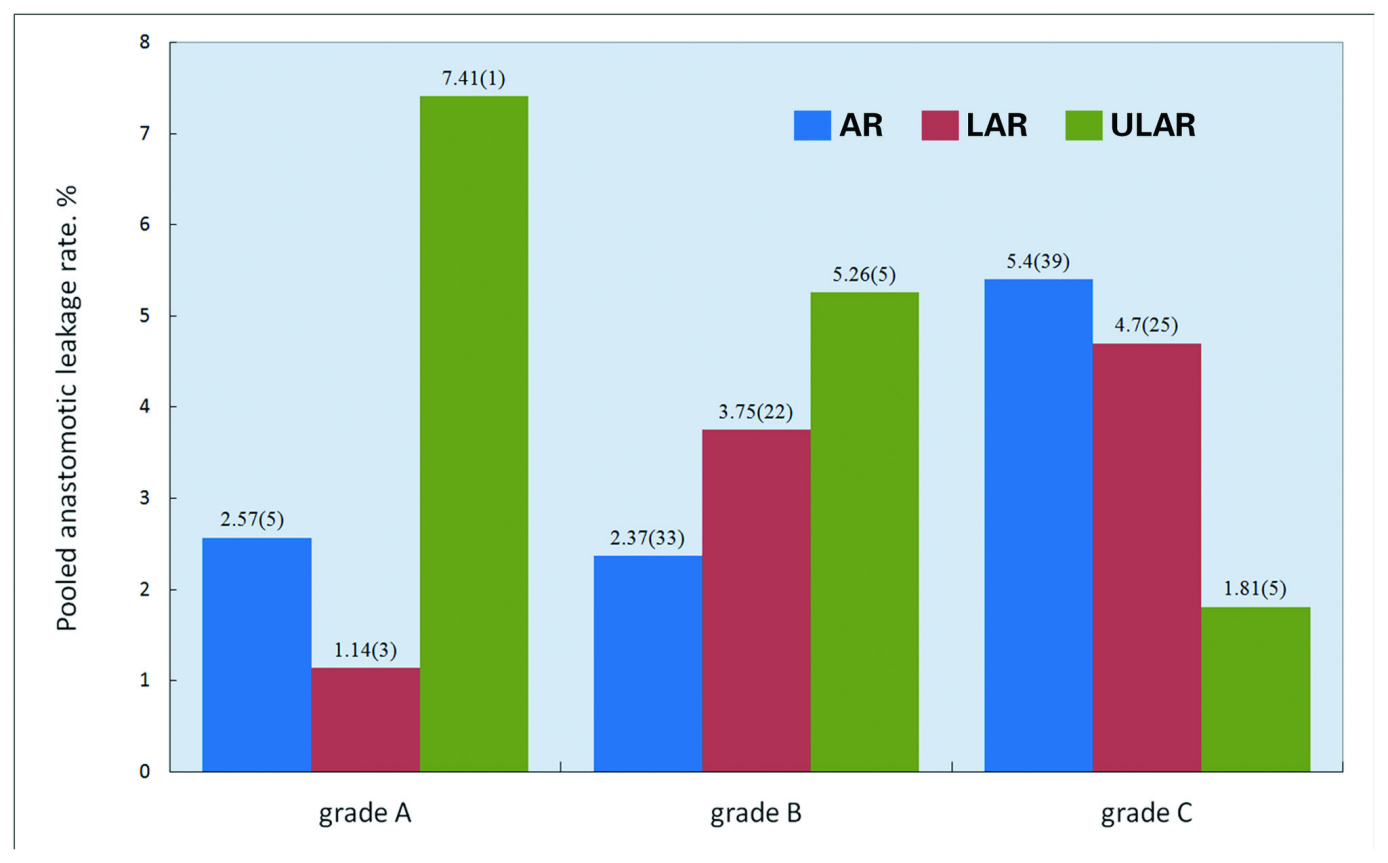
Pooled AL rates of each grade in AR, LAR, and ULAR (the number of reports are presented in brackets).

Five studies on ULAR matched the inclusion criteria, which had a total population of 551 patients. ULAR was described as intersphincteric resection or resection in the ultralow rectal cancer in these studies. A total of 41 leaks were confirmed; the pooled overall rate of AL was 7.44% (which ranged from 4.65% to 12.50%). The pooled rates of Grades A, B, and C leakages were 7.41% (2/27 from one study), 5.26% (29/551 from all five studies), and 1.81% (10/551 from all five studies), respectively.

The pooled overall rates of AL in AR and LAR were similar (8.58% versus 8.88%) and were both a little higher than that in ULAR (7.41%). The pooled rates of AL for each grade in AR, LAR, and ULAR are shown in Figure 6. Unfortunately, only very few studies (only nine studies in all the three groups) provided data for the Grade A leakage; thus, we could not make a substantial analysis of the trend of AL in the three groups from the few available data. However, the data for Grades B and C were sufficient to be credible. The pooled rate of Grade C leakage in the AR studies was the highest (AR > LAR > ULAR). On the other hand, the pooled rate of Grade B was the lowest in AR (AR < LAR < ULAR).

The data of the temporary DS in the initial operation were also extracted and compared. The pooled rates of DS in the 29 AR studies, 21 LAR studies, and 4 ULAR studies were 30.11% (6,024/20,006), 40.72% (1,643/4,035), and 63.44% (295/465), respectively. The three results (AR < LAR < ULAR) also showed the same trend as that of the Grade B leakage and an opposite trend with that of the Grade C leakage.

### Pooled rate of AL with each grade after AR in different countries

The included AR reports consisted of 18 reports from Asia [2,14-30], 21 from Europe [3, 4, 6, 31-48], and 1 from the US [49]. The pooled regional AL rates were 5.43% in Asia (511/9,406), 11.09% in Europe (1,526/13,755), and 4.26% in the US (48/1,127). The difference between the US and Asia in the overall AL rate after AR was not significant (*P* = 0.097). However, the rate was significantly higher in Europe than in the US and Asia (both *P* < 0.001). The same situation was true for the regional rate of Grades B and C leakages (Table 1).

## Discussion

AL has been well known as a predominant cause of morbidity and mortality after AR [33,53]. In addition, some authors also reported that leakage impaired long-term prognosis of patients with rectal cancer, in addition to the adverse effect on late functional results, particularly when operative re-intervention was required [12,68,79-81]. The administration of adjuvant chemotherapy may be prevented or delayed for the occurrence of AL in these patients, which is probably associated with poor oncological outcome. Therefore, the incidence of AL after AR was considered an essential measure to evaluate the clinical value of the different operative and perioperative interventions and, hence, is selected frequently as a primary end point in clinical trials. However, because of the lack of objective and easily applicable definitions of AL, the results among studies did not allow simple comparison and, therefore, clear conclusions as to which type of operative and perioperative management should be preferred in daily practice were hindered.

To standardize the reporting of clinical studies, ISGRC proposed a generally acceptable definition and grading of the severity for AL in 2010 [5], which have been adopted by clinicians in reporting their studies and helped readers compare the results of different reports. This definition comprised all types of leakages ranging from an asymptomatic leakage to a leakage resulting in life-threatening conditions, and the grading system was defined according to the clinical management of AL, which can be applied easily within the routine clinical care and is likely to be accepted and applied in future studies.

AL is classified as Grade A when not associated with clinical symptoms, which is commonly detected by contrast enema studies during routine diagnostic workup before the closure of a temporary ileostomy/colostomy [82]. We regret to find that Grade A leakage cases were only extracted from five reports on AR in our review. Because of the almost harmless character of this type of AL, most authors in the literature may not have required routine imaging studies, which made the radiologic AL difficult to discover. Moreover, when occasionally found by imaging, conservative treatment such as prophylactic antibiotic therapy might be needed instead of simple fasting and observation, even when no symptom is observed in the patient.

AL is classified as Grade B when the patient’s clinical condition requires an active therapeutic intervention that can be managed without operative re-intervention. Patients suffer from mild to moderate distress, characterized by abdominal and/or pelvic pain and possible abdominal distension. Pelvic drains may discharge turbid/purulent or fecal fluid, although the presence of this finding depends on the size of the leakage and is alleviated in patients with DS. Moreover, patients with Grade B leakage may complain of turbid/purulent rectal or vaginal discharge.

AL is classified as Grade C when the patient is quite ill and requires operative relaparotomy. These patients usually have abdominal pain and fever and subsequently develop signs of peritonitis (tenderness to palpation, abdominal wall rigidity, and tachycardia). If operative re-intervention to control the septic source is delayed or not performed, the clinical condition of these patients could deteriorate and ultimately results in sepsis with clinical signs of hypothermia, leukopenia, and organ failure. In this thorough overview of the studies, we extracted a considerable pooled rate of 5.40% for Grade C leakage, which means that 63.08% of AL patients needed operative re-intervention. Moreover, 89.55% of these patients with Grade C leakage needed temporary or permanent diverting-stoma construction.

Interestingly, although the difference in the overall rates of AL among the reports on AR, LAR, and ULAR was not very large, an opposite trend of the pooled rate of Grades B and C leakages was observed in these three groups, as revealed in the present systematic review (Grade C rate in AR > Grade C rate in LAR > Grade C rate in ULAR and Grade B rate in AR < Grade B rate in LAR < Grade B rate in ULAR). This results means that more ALs required operative re-intervention in the reports on AR than those on LAR and ULAR. We believed that the difference in the rate of protective DS in the initial operation among the reports on AR, LAR, and ULAR could explain this interesting situation because primary DS can effectively reduce the risk of AL that requires relaparotomy, as reported by other authors [3,43,83,84]. Our findings in this review revealed the following: the pooled rate of primary DS in AR < the pooled rate of DS in LAR < the pooled rate of DS in ULAR.

The pooled rate of AL in the European countries was much higher than those in the Asian countries and the US (*P* < 0.001). We cannot determine whether any ethnic or regional differences exist in the actual incidence of AL in these populations. Of course, the differences in the language background and the indications for neoadjuvant chemoradiation therapy between the European and Asian countries cannot be ignored [85,86].

It would be informative to know, what proportion of surgery was performed by colorectal surgical specialists. We failed to give this data because most of our selected publications didn’t give the proportion of colorectal surgical specialists except for those involve influencing factors analyzing. But many studies in current literature demonstrated that leak rates were thought to be lower in specialist, rather than general surgical hands. Our previous work also supported this point, which showed that the rate of leak in colorectal surgical specialists group and general surgeons group were 3.9% and 11.3% (P=0.031), respectively [14].

The other limitation of this article is the sourcing of the publications. All abstracts were retrieved from Medline, and non-English language papers were excluded in the final analysis. A formal meta-analysis will provide more powerful evidence. The methodology used in the present study was not as powerful as a meta-analysis. However, our systematic review provides the summarized data directly from the original publications on the AL rate and on grading.

## Conclusion

We have extracted a significant pooled rate of AL that required relaparotomy following AR for rectal cancer, which was higher than those in LAR and ULAR. The higher rate of protective DS in LAR and ULAR might be the cause of this difference. Compared with past situations when authors used their own definitions and grading in the studies, we now have a generally accepted definition and grading of the severity of AL. This new grading system is simple that ALs of each grade could be easily extracted from past publications. Therefore, this simple classification is likely to be accepted and applied in future studies.
